# Rotameric Heterogeneity of Conserved Tryptophan Is Responsible for Reduced Photochemical Quantum Yield in Cyanobacteriochrome Slr1393g3

**DOI:** 10.1002/cphc.202400453

**Published:** 2024-11-12

**Authors:** Lisa Köhler, Florian Trunk, Valentin Rohr, Tobias Fischer, Wolfgang Gärtner, Josef Wachtveitl, Jörg Matysik, Chavdar Slavov, Chen Song

**Affiliations:** ^1^ Institut für Analytische Chemie Universität Leipzig 04103 Leipzig Germany; ^2^ Institut für Physikalische und Theoretische Chemie Goethe-Universität Frankfurt 60438 Frankfurt am Main Germany; ^3^ Department of Chemistry University of South Florida 33620 Tampa United States of America

**Keywords:** NMR spectroscopy, Isotopic labeling, Isomers, Photochemistry

## Abstract

The red/green cyanobacteriochrome (CBCR) slr1393g3 exhibits a quantum yield of only 8 % for its forward photoconversion, significantly lower than other species from the same CBCR subfamily. The cause for this reduced photoconversion is not yet clear, although in the related NpR6012g4 dark‐state structural heterogeneity of a paramount Trp residue has been proposed to cause the formation of nonproductive subpopulation. However, there is no such information on the equivalent residue in slr1393g3, W496. Here we use solid‐state NMR to explore all possible sidechain rotamers of this Trp residue and their local interactions at the atomic level. The indole nitrogen (N*ϵ*1) is used as an NMR probe, achieved by site‐specific ^15^N‐indole labeling of a quadruply Trp‐deleted variant and trehalose vitrification technique. The data reveal a set of seven indole rotamers of W496 with four distinct environments for the N*ϵ*1‐H group. Only a minority population of 20 % is found to retain the π‐stacking and hydrogen‐bonding interactions with the chromophore in the dark state that has been assigned to account for complete forward photoconversion. Our results demonstrate the direct role of W496 in modulating the forward quantum yield of slr1393g3 via rearrangement of its sidechain rotameric conformations.

## Graphical abstract

The NMR‐based evidence for structural heterogeneity at a conserved Trp residue in the CBCR slr1393g3 dark state is clearly set forth, by selectively ^15^N‐labeling its indole nitrogen in a quadruply Trp‐deleted (Y^2^F^2^) variant. Four types of indole rotameric conformations with distinct local interactions are revealed. A minority conformation retaining interactions with the chromophore (20 %) is assigned to account for complete forward photoconversion.

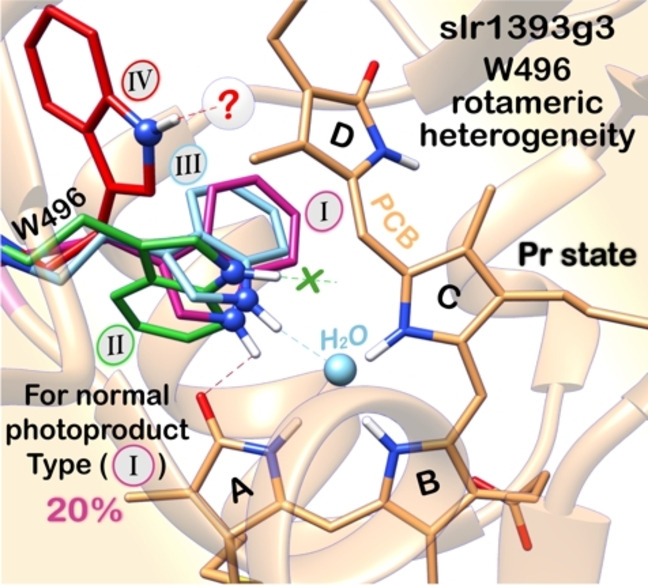

## Introduction

Most living organisms have developed light‐detection systems in order to identify intensity, spectral composition, duration, direction, or even the polarization of light. This capability derives from photosensory proteins which incorporate and bind a small ligand (‘chromophore’) with a characteristic π‐conjugated system.^[1]^ Phytochromes constitute a large and diverse superfamily of bilin‐binding c**G**MP phosphodiesterase/**A**denylyl cyclase/**F**hlA (GAF) domain‐containing photoreceptors that were divided into three subgroups, according to domain architecture.[^2^, ^3^, ^4^] Cyanobacteria contain not only the canonical and ‘knotless’ phytochromes, but also examples of ‘GAF‐only’ CBCRs. Remarkably, a single bilin‐binding GAF domain is sufficient for a fully functional CBCR photocycle.^[4]^ Despite the compact size, the CBCR‐GAF domains exhibit a significant variation in absorption covering the entire UV‐to‐visible spectrum with binding four types of bilin chromophores such as biliverdin (BV),[^5^, ^6^] phytochromobilin (PΦB),^[7]^ phycocyanobilin (PCB),[^8^, ^9^, ^10^, ^11^] and phycoviolobilin (PVB).[^12^, ^13^, ^14^] Given the distinctive spectral tuning mechanisms, four subfamilies of CBCRs have been described,^[4]^ among which the red/green‐absorbing species have received the most attention as molecular templates for further understanding of structure–function relationship in phytochromes in general,[^15^, ^16^, ^17^, ^18^, ^19^, ^20^, ^21^, ^22^, ^23^, ^24^, ^25^, ^26^] and development of optogenetic and bioimaging tools.[^5^, ^27^, ^28^, ^29^, ^30^, ^31^]

**Figure 1:**
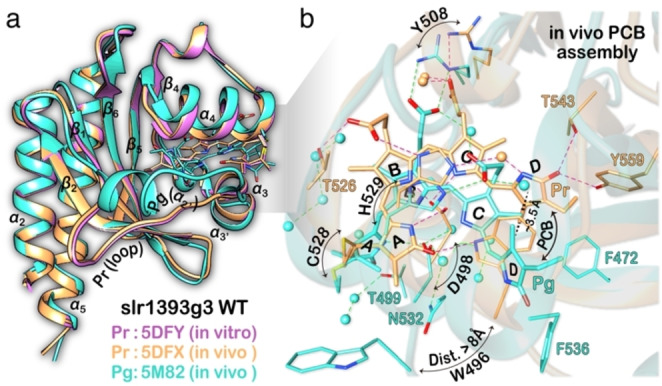
Crystal structures of CBCR slr1393g3 assembled with PCB. a) Superposition of the Pr state of the in vitro‐assembled protein (lilac, PDB ID 5DFY) and the in vivo‐assembled counterpart in both Pr (apricot, 5DFX) and Pg (turquoise, 5M82) states. The two Pr‐state structures are nearly identical to each other with an RMSD value of 0.13 Å over 158 C*α* atoms. The Pr → Pg photoconversion is associated with a conformational switch from a loop (between *β*_2_ and *α*_3_) in Pr to a two‐turn helix in Pg (*α*_2’_). b) The chromophore‐binding pocket of the in vivo‐assembled protein in both states with their respective protein–chromophore hydrogen‐bonding networks (dashed lines). Water molecules are shown as spheres and colored accordingly. The W496 indole ring undergoes a large displacement upon photoconversion.

Red/green CBCRs represented by AnPixJg2,^[23]^ NpR6012g4,^[24]^ as well as slr1393g3 from *Synechocystis* 6803 (Figure 1a) exhibit a red‐absorbing Pr dark state similar to that of canonical phytochromes but a hypsochromically shifted green‐absorbing Pg photoproduct. In the native form, the photoproduct of slr1393g3 is characterized by a photochromic shift of 113 nm to shorter wavelengths (*λ*_max_=649 and 536 nm for Pr and Pg, respectively). The phototransformations between these two states add further color complexity: in both reaction pathways, yellow‐ and orange‐absorbing intermediates have been characterized.[^22^, ^32^] The shortened conjugation length of the chromophore evoked by the twisted ***D***‐ring geometry has been proposed to explain the spectral shift of Pg photoproduct.^[20]^ Besides the ‘***D***‐ring control’ hypothesis, a polaronic defect of the core conjugation formed by the inner rings ***B*** and ***C*** plays a vital role in tuning the spectral properties of various slr1393g3 photocycle states.^[22]^

Like phytochromes, red/green CBCRs are found to be structurally, photochemically, and spectrally heterogeneous.[^18^, ^22^, ^24^, ^33^, ^34^, ^35^, ^36^, ^37^, ^38^, ^39^, ^40^, ^41^, ^42^, ^43^] Here, the structural heterogeneity refers to both sidechain rotamers of certain amino acids varied with the protonation and tautomeric states[^24^, ^33^, ^34^, ^42^] and the chromophore conformers considering geometry, charge distribution patterns, and surrounding protein–chromophore interactions.[^20^, ^40^, ^41^, ^42^] Photochemical and spectral heterogeneity includes excited state subpopulations (e. g., photoactive vs fluorescent) decaying on different time scales[^44^, ^45^, ^46^, ^47^] and also subpopulations observed in absorption and fluorescence excitation spectra, whose relative ratio varies with temperature and pH.[^16^, ^24^, ^41^] Recently, the rotameric structures of the Asp and Trp residues near the chromophore in the NpR6012g4 dark state have been linked to subpopulations with distinct absorption and fluorescence properties.^[24]^ This partially conserved Trp residue in red/green CBCRs (e. g., W496 in slr1393g3, as in Figure 1b) shifts radically upon photoconversion, such that the indole ring is no longer π‐stacked with the ring ***D*** as seen in the Pr dark state, but is brought closer to the ethylidene sidechain of ring ***A***. This leads to a water influx and the formation of a solvated binding pocket for the photoproduct chromophore (Figure 1b). Although this intriguing Trp residue is dispensable for red/green photocycles,[^10^, ^23^, ^51^] it modulates the electronic transition of the chromophore in the dark state and tunes the fluorescence of red/green CBCRs.[^48^, ^49^, ^50^] However, in the case of slr1393g3, despite evidence of a structurally heterogeneous chromophore in all photocycle states,^[23]^ no alternative sidechain conformations at W496 and other key residues interacting with the chromophore have been traced in the corresponding crystal structures^[25]^ and in recent spectroscopic and theoretical studies.[^15^, ^16^, ^20^, ^23^, ^49^, ^50^] Moreover, detailed characterization of presence of spectral heterogeneity in this protein has not been performed.

This study was therefore undertaken to assess whether W496 serves as a site of structural variability in the slr1393g3 dark state and its correlation with the unusually low quantum yield for the Pr → Pg forward reaction of 8 %,[^15^, ^52^] relative to 40 % for that of NpR6012g4.^[36]^ We here employ solid‐state magic‐angle spinning (MAS) NMR spectroscopy to investigate rotameric conformations of this specific lid Trp residue and the associated changes in hydrogen‐bonding properties. This is achieved by ^15^N labeling of the indole nitrogen atom (W496N*ϵ*1) upon removal of the other four residual Trp residues in the sequence — W470, W483, W553, and W567 — via site‐specific mutagenesis into either Tyr or Phe (W470Y/W483Y/W553F/W567F, see Experimental Procedures in SI for details). The resulting quadruply Trp‐deleted (Y^2^F^2^) variant was then embedded into trehalose glasses (TGs).[^22^, ^53^, ^54^, ^55^, ^56^] The use of highly integrated glassy system enables an overview on most abundant rotameric structures of the target residue, e. g., W496 in this case, without perturbations in the native protein structure.[^22^, ^53^] Trehalose is well documented to preserve structural and functional integrity of labile proteins with respect to freeze‐thawing and freeze‐drying procedures, and moreover, thermal denaturation of the TG‐embedded proteins is inhibited for periods as long as months at room temperature.[^22^, ^53^, ^56^, ^57^] Our data provide an accurate description of possible rotameric structures retained by W496 in the slr1393g3 dark state which form the basis for an improved understanding of the three‐ to four‐fold observed reduction in the forward quantum yield for this protein compared to other related species, thus improving the use of CBCRs for synthetic biology.

## Results and Discussion

### Substitution of W496 Promotes Spectral Heterogeneity in the slr1393g3 Dark State

**Figure 2:**
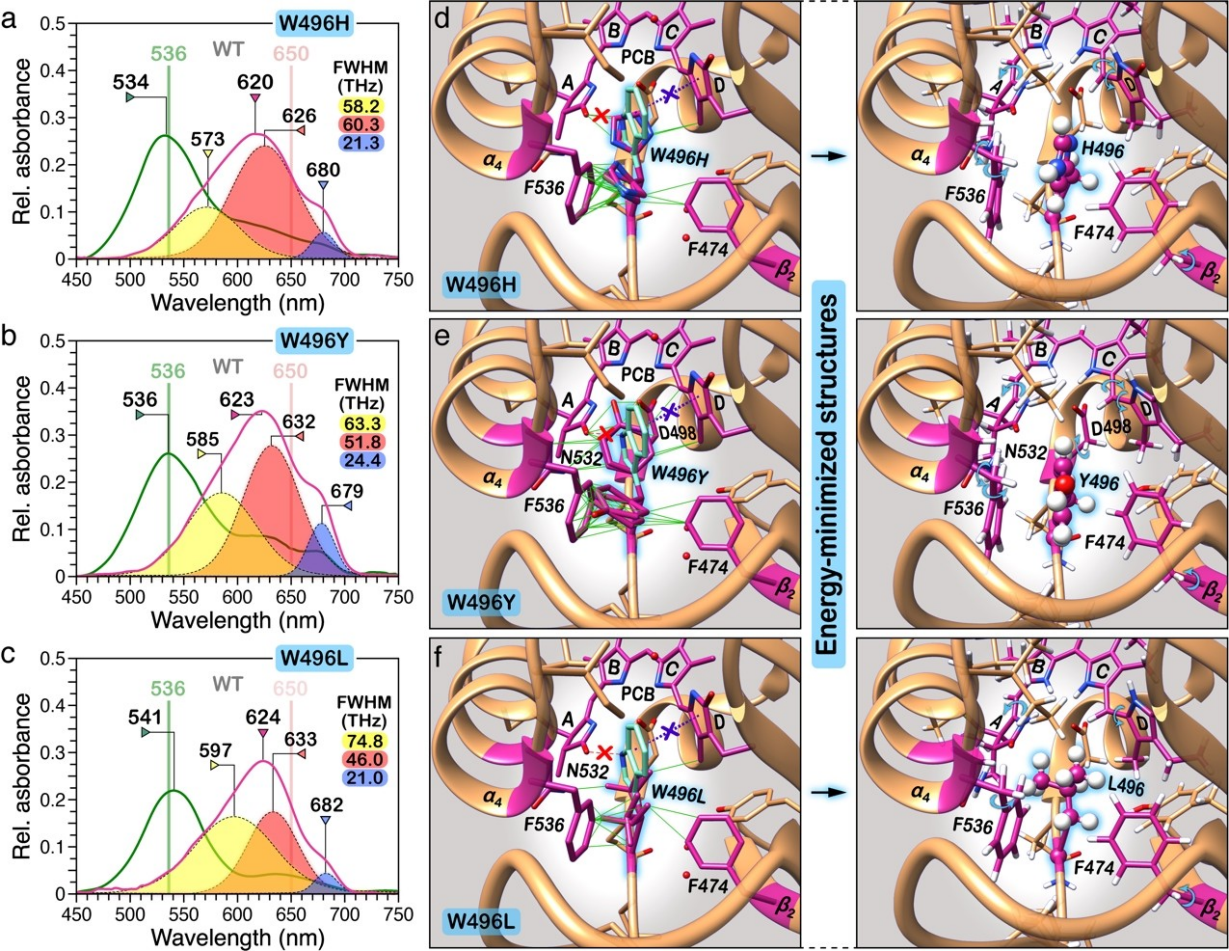
Absorption and structural characteristics of three W496 variants from slr1393g3. UV‐vis absorption spectra of a) W496H, b) W496Y, and c) W496L in their respective dark states (pink) and photoproducts (green). The *λ*_max_ values of the WT (indicated by vertical bars) are given as reference. Deconvoluted absorption bands are shaded. The *R* ^2^ values of all fits are >0.99. The FWHM values of the obtained dark‐state spectral components are given in units of terahertz (THz). Structural representation of the sidechain conformations of d) W496H, e) W496Y, and f) W496L that were clustered into rotamers with an overall probability of >70 % (for the four most probable sidechain conformations) using the Dynameomics rotamer library (UCSF Chimera). For comparison, the W496 residue (light teal) is kept which locates in the unconstructed loop between *β*_2_ and *α*_3_. The potentially clashing pairs generated upon mutation are highlighted by the green pseudobonds. (*Right panel*) To eliminate the steric clash, the structures were subjected to energy minimization by employing the conjugate gradient algorithm and Amber force field (AMBER ff14SB) through UCSF Chimera. Specifically, the energy minimization was performed using 500 steepest descent steps with a 0.02 Å step size and an update interval of 10. The minimization needs to have all protons in place and only the most probable conformation of the substituted residue is shown. Modifications of the torsional angles of both the neighboring residue sidechains and the two outer rings of the chromophore (***A*** and ***D***) are indicated by cyan arrows.

We first examine whether the lid Trp fulfils a similar role in tuning the stability and spectral properties of the slr1393g3 dark state as seen in NpR6012g4^[51]^ using site‐specific mutagenesis. W496 in slr1393g3 was substituted by His and Tyr maintaining the aromatic character and also by aliphatic Leu with a smaller sidechain. All three variants (W496H/Y/L) investigated here exhibited a similarly blue‐shifted dark state (26–30 nm) with little or no effect on the photoproduct absorption maximum (Figure 2a–c), regardless of the steric demand or the physicochemical properties of the substituted residues. Similar effects have been also noted in the equivalent variants of AnPixJg2^[23]^ and NpR6012g4,^[51]^ supporting the role of the lid Trp preferentially for the formation and stabilization of the dark state.

Intriguingly, the band analysis revealed a major broadening of the Q‐bands (the bandwidth expressed as full width at half maximum, FWHM) of the three variants relative to that of WT (FWHM ranging between 46.0 and 60.3 THz for variants and of 30.7 THz for WT), implicating a less ordered chromophore. Indeed, line‐fitting of any dark‐state spectrum of these variant proteins reveals at least three spectral components with different band widths (Figure 2a–c). Besides the central band ranging from 626–633 nm, the short‐wavelength population absorbing in the yellow‐to‐orange region of the spectrum becomes more abundant, particularly noticeable for W496L (Figure 2c). Similar accumulation of orange‐absorbing population was seen upon substitutions of W496‐homologous residue in the NpR6012g4 dark state which has been found to be photochemically inert, arising due to the structural heterogeneity of this Trp residue.^[24]^ Surprisingly, the three variant proteins exhibited a long‐wavelength shoulder in the dark state whose apparent maximum at around 680 nm matches those of the free protonated bilin chromophores in solution, having less steric constrains on the conjugation chain.^[58]^ This band, assigned to be the third population of the dark state, is not fully convertible upon photoconversion (Figure 2a–c), even with exhaustive illumination from LEDs at 675±17 nm. Such a long‐wavelength population is not seen in the WT absorption spectrum,^[24]^ and, moreover, the FWHM band width is two‐ to three‐fold smaller than that of WT. These observations implicate a well‐defined chromophore environment for this minority population with most likely increased protein–chromophore interactions. A similar long‐wavelength shoulder in the dark state was also seen in the W496I variant,^[10]^ but no such effect was detected upon introducing Val or Ala at this position in NpR6012g4.^[51]^ We thus propose that the presence of this population absorbing at around 680 nm correlates with steric demand of the substituted residue, an effect that probably is absent in the NpR6012g4 variants due to differences in protein–chromophore interactions around ring ***D***.

Unlike the dark state, substitutions of W496 had impact neither on the absorption maxima (Δ*λ*_max_≤5 nm) nor on the lineshape of the Pg photoproduct (Figure 2a–c), even though the substitutions involve huge deviations in chemical character. Despite yielding a normal photoproduct, the photoconversion of all three variants is incomplete with residual red absorption (illuminated with 650±17 nm light). Similar effect was detected upon introducing Ala at this position in NpR6012g4 (W655A).^[51]^ These observations demonstrate that such substitutions for W496 result in the formation of side populations in the slr1393g3 dark state without significant effects on the spectral properties of the photoproduct.

We next analyzed the changes in the sterically favored geometry adopted by the chromophore and the local environment in the protein moiety upon substitutions of W496. For each variant, the four most probable sidechain conformations of the substituted residue were chosen from the rotamer library (UCSF Chimera)^[59]^ with an average probability of ≥14 % (Figure 2d–f). Common to all refined rotameric structures obtained after energy minimization is the apparent loss of both aromatic stacking onto the bilin ring ***D*** and hydrogen‐bonding with the ring ***A***. Moreover, severe steric clashes in the variants have been minimized, particularly between the chromophore and a number of residues nearby including F474 (‘*β*_2_ Phe’), F536 (‘helix Phe’), and N532 from the *α*_4_‐helix as well as the central D498 within the conserved DXXLQ motif. Possible structural modifications might involve the twisting/untwisting of both outer rings ***A*** and ***D*** of the chromophore and the sidechain rotamerization of those residues (Figure 2d–f). Intriguingly, the Tyr or His residues in place of the lid Trp appear not to be in a suitable position to provide an alternative hydrogen bond donor to the chromophore which presumably determines a looser, less energetically favorable packing than in WT. These two variants form heterogeneous mixtures in the dark state comprising a major red‐absorbing population (around 620 nm) and two minor populations with peak absorptions at shorter and longer wavelengths (around 580 and 680 nm, respectively, Figure 2a and b). The W496Y variant exhibited an increase in the short‐wavelength population relative to that of W496H which becomes even the majority dark‐state population in the W496L variant (Figure 2c). The relative ratio of the three dark‐state populations seems to be closely correlated with the size, charge, and hydrophobicity of the substituted residue, emphasizing the essential role for W496 in tuning stability and spectral properties of the dark state.

### Replacement of the Four ‘non‐lid’ Trp Residues with either Tyr or Phe has Little Effect on the Steady‐state and the Dynamic Features

**Figure 3:**
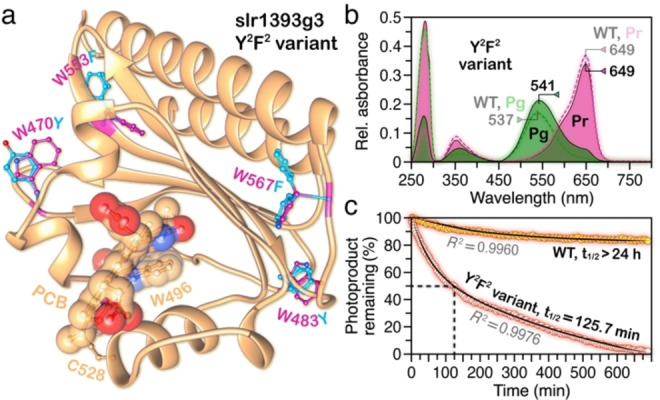
Absorption properties and thermal stability of the slr1393g3 quadruple Y^2^F^2^ variant. a) Ribbon diagram of the slr1393g3 mainchain in the Pr dark state (5DFX). The PCB chromophore and the lid Trp residue W496 are highlighted in yellow spheres. Other four residual Trp residues in the protein are shown in stick mode and colored magenta. The two most probable sidechain rotamers of each introduced residue (Tyr for W470 and W480 and Phe for W553 and W567) are clustered with an average probability of 0.19 calculated using the Dynnameomics rotamer library (UCSF Chimera) and colored teal. b) UV‐vis absorption spectrum of the Y^2^F^2^ variant in Pr and Pg (colored pink and chartreuse, respectively) in comparison with the WT (dashed curves). c) Thermal reversion of the variant in comparison with the WT. The *R* ^2^ values of both fitting curves (in black) are >0.99.

We consider whether the lid Trp W496 is also a site of structural heterogeneity in the slr1393g3 dark state, as has been previously noted for NpR6012g4.^[51]^ We therefore generated a quadruple variant replacing all four residual Trp residues (W470, W483, W553 and W567) in slr1393g3 by Tyr and Phe (Figure 3a) to allow site‐specific isotope labeling of W496 for solid‐state NMR studies. Substitutions of Tyr for W470 and W483 mostly retain the original hydrogen‐bonding networks, and substitutions of Phe for W553 and W567 are designed to enforce similar steric constraints on neighboring residues. The W470Y/W483Y/W553F/W567F (Y^2^F^2^) variant exhibited a minor photoproduct red‐shift of only 4 nm relative to WT with no effects on the dark‐state absorption and emission (Figure 3b). Photoconversion of the quadruply Y^2^F^2^ variant is incomplete with residual red absorption at 649 nm in the Pg photoproduct (upon exhaustive illumination from LEDs at 650±17 nm) which could arise from the extended Pg absorption spectrum and the higher quantum yield of the reverse Pg → Pr reaction.

**Figure 4:**
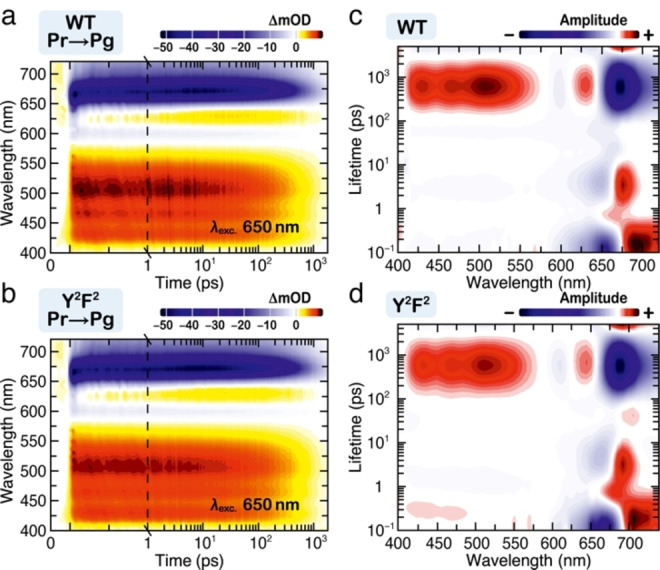
Ultrafast excited‐state dynamics of the slr1393g3 WT and Y^2^F^2^ variant. (a and b) Transient absorption data of the forward (Pr → Pg) dynamics of the WT (a) and the Y^2^F^2^ variant (b). (c and d) Corresponding LDMs obtained from lifetime distribution analysis of the WT (c) and the variant (d). The ultrafast data are shown after excitation of the Pr state at 650 nm.

**Figure 5:**
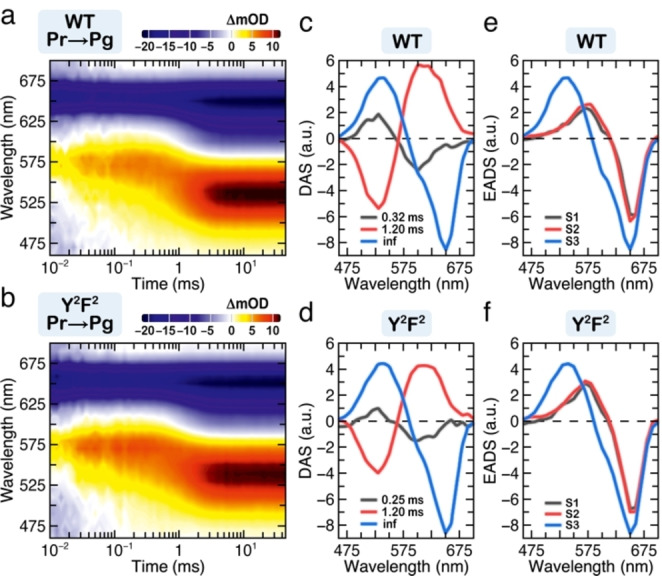
Millisecond dynamics of the slr1393g3 WT and Y^2^F^2^ variant. (a and b) Millisecond forward dynamics (Pr → Pg) of the WT (a) and the variant (b). (c and d) Corresponding DAS data. (e and f) Corresponding EADS data. The millisecond dynamics were obtained after excitation of the Pr state at 650 nm.

To ensure that the photochemistry of the Y^2^F^2^ variant remains unchanged, we performed femtosecond transient absorption (TA) experiments and millisecond flash photolysis and compared the data to those of the WT (Figures 4 and 5). The Pr → Pg TA data of both WT and the Y^2^F^2^ variant as well as the lifetime density maps (LDM) were obtained from the lifetime distribution analysis using OPTIMUS (https://optimusfit.org/)^[60]^ (see Data analysis in SI for details). The TA data of the two proteins are essentially identical (Figure 4a and b): they consist of a broad excited state absorption (ESA, positive difference signal in the 400–650 nm range). In the 550–650 nm range, however, the ESA is superimposed with the ground state bleach (GSB, negative difference signal). Above 650 nm, where the steady‐state fluorescence is located, we observed stimulated emission (SE, negative difference signal). The LDMs reveal further kinetic details (Figure 4c and d). In accordance with our previous assignment,^[15]^ above 630 nm, we observed a clear distribution of positive and negative amplitudes with ~100 fs lifetime which are associated with a shift in the SE band due to the departure from the Franck–Condon region. This is followed by a series of alternating in amplitude lifetime distributions (~300 fs to ~40 ps) in the 600–680 nm range. These are not complemented by a characteristic for excited state decay positive amplitude distribution in the ESA range (<570 nm), and thus can be assigned to conformational dynamics of the chromophore on the excited state potential energy surface accompanied by reorganization of its local environment in the pocket.[^37^, ^43^, ^61^, ^62^, ^63^] The dominant features in the LDM are found in the 100–2000 ps range: i) the positive amplitude distribution (400–550 nm) describes the decay of the ESA, while ii) the negative amplitude distribution >650 nm describes simultaneously the decay of the SE, the partial recovery of the GSB, as well as the rise of the primary photoproduct. The limited detection window in our experiments does not permit complete observation of the primary photoproduct formation. Nevertheless, the positive amplitude distribution on the upper limit of the LDMs indicates such formation.

To further verify that the photochemistry remains conserved upon Y^2^F^2^ substitutions, we investigated the millisecond dynamics using laser flash photolysis (Figure 5). The results demonstrate that the Pr → Pg millisecond dynamics is also not affected. The data consist of a negative signal (630–680 nm) assigned to the GSB as well as two different positive signals associated with photoproduct absorption (PA). The first, peaking at 570 nm, is observed at early delay times (0.01–1 ms), while the second at 540 nm rises at ~1 ms and remains until the end of the measurement timescale. Global target analysis (GTA) using a sequential kinetic model^[60]^ was applied to reveal the kinetic and spectral details. Three lifetimes were required to adequately describe the datasets. The first lifetime (0.32 and 0.25 ms in the WT and the Y^2^F^2^ variant, respectively) describes the build‐up of the orange‐absorbing intermediate at 570 nm which subsequently decays with a lifetime of 1.2 ms forming the final photoproduct. Comparing both the ultrafast and the millisecond data, we observed no significant difference between the Y^2^F^2^ variant and the WT. Our results therefore demonstrate that the four Trp residues — whose sidechains locate at least 9.5 Å away from the chromophore (Figure 3a) — are not critical determinants for modulating the spectral and kinetic properties of the slr1393g3 Pr dark state. Moreover, the reverse Pg → Pr reaction of the WT and its Y^2^F^2^ variant on the ultrafast timescale show far‐reaching similarities, except for a slightly accelerated intermediate decay of the last transition to form the Pr dark state (see Data analysis in SI for a detailed description).

Surprisingly, such substitutions accelerate the thermal reversion of the photoproduct by two orders of magnitude relative to WT (*t*_1/2_=125.7 min for Y^2^F^2^ variant vs days for WT, Figure 3c), even though the forward reaction is hardly affected. In this case, modifications induced by the substitution on the intimate contacts between protein and chromophore can be precluded. On the other hand, the thermal reversion kinetics does not depend on the interaction between the chromophore and its binding pocket, but rather on larger scale conformation changes in protein.[^10^, ^64^] Thus, these surface Trp residues have a stabilizing function for the protein structure of the photoproduct, although none of them directly interact with the chromophore.

### W496 Exhibits a Multitude of Sidechain Rotameric Structures in the slr1393g3 Dark State

**Figure 6:**
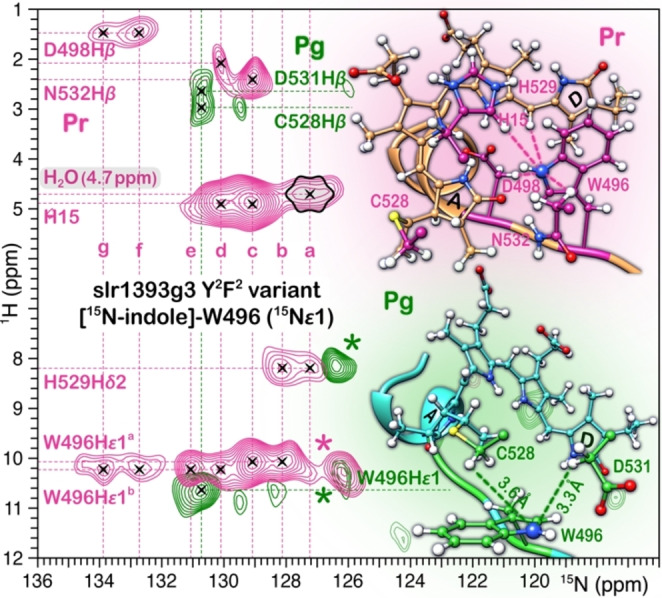
Selective observation of interfacial and intramolecular ^1^H contacts of the [^15^N‐indole]‐labeled W496 (^15^N*ϵ*1) in the Y^2^F^2^ variant. Stacked ^15^N–^1^H correlation contour plots of the Pr dark state (pink) and Pg photoproduct (green). The splitting components of ^15^N signals are indicated by vertical dashed lines and labeled with letters. The ^1^H contacts are indicated by the horizontal dashed lines. Structural views showing ^1^H contacts of the W496N*ϵ*1 atom (marked as blue sphere) observed in correlation spectra (highlighted by the dashed lines). The corresponding interatomic distances are extracted from the Pr 5DFX and Pg 5M82 structures.^[25]^ Correlations marked with an asterisk indicate amide nitrogens of protein backbone originating from ^15^N in natural abundance.

Given that our results indicate that substitutions of four non‐lid Trp residues have little or no effect on the local chromophore environment in the dark state, we sought to characterize the structural heterogeneity of W496 at the atomic level using solid‐state NMR. The ^15^N‐labeled indole of W496 (N*ϵ*1) in the quadruply Y^2^F^2^‐substituted variant was chosen as an NMR probe. The advantage of utilizing the ^15^N nucleus lies in a much more simplified NMR spectrum, i. e., only one resonance per indole rotameric conformer appears. We first performed a ^15^N–^1^H HetCor experiment to observe correlations between W496N*ϵ*1 nitrogen and adjacent protons (Figure 6). Strikingly, the N*ϵ*1 atom in the dark state exhibited a set of seven ^15^N resonances (labeled as W496N*ϵ*1^a–g^, Figure 6 in pink) with a wide dispersion of 6.7 ppm, indicative of sidechain rotameric heterogeneity because the ^15^N chemical shift of the indole nitrogen is known to be largely determined by the geometry of the indole ring itself rather than the surrounding environment. The resonance splitting is also evident in the ^1^H dimension for the proton directly‐bonded to the N*ϵ*1 nitrogen (H*ϵ*1), potentially implicating variations in the local environment of individual indole rotameric structures. Further support for this interpretation stemmed from the complete assignment of the interfacial ^1^H residue/chromophore contacts of W496N*ϵ*1 atom in which at least five types of nearest ^1^H contacts were identified in the seven ^15^N slices (Figure 6). For example, the ^1^H site at 4.9 ppm was assigned to H15 based on the similar shift values for this proton in other related CBCRs.[^26^, ^42^, ^65^] This ^1^H slice exhibited two correlations from the N*ϵ*1^c^ and N*ϵ*1^d^ atoms, demonstrating the proximity of the methine H15 atom and W496N*ϵ*1 in these two rotameric subpopulations. Both interfacial contacts are consistent with the π‐stacking distance of ~3.5 Å between the W496 indole and the chromophore ring ***D*** in slr1393g3 WT (Figure 1b) as well as the ‘Trp‐in’ conformation for the W496‐homologous residue in the NpR6012g4 dark state.^[24]^ However, no such correlations could be detected in the other five N*ϵ*1 slices. The absence of this distance restraint characteristic of the ‘Trp‐in’ rotamer provides good evidence that the indole rotated away from ring ***D***, resulting in a larger separation between N*ϵ*1 and H15 beyond the 3.7 Å‐detection limit for N−H interactions, experimentally determined with an LG–CP contact time of 900 μs.[^40^, ^42^]

We also noted that the intermolecular contacts between N*ϵ*1 and its bonded proton (H*ϵ*1) are present in six out of seven rotameric structures but absent for the N*ϵ*1^a^ species with *δ* ^N^ of 127.2 ppm (Figure 6). The simplest interpretation of this observation is that the N*ϵ*1^a^ resonance has a unique ^1^H→^15^N cross‐polarization (CP) kinetics, arising from the high flexibility of this conformational population,[^39^, ^66^] consistent with a recent *ab initio* study of AnPixJg2.^[34]^ Alternatively, we considered the possibility of an only partial occupancy of the proton at N*ϵ*1 or a significantly stretched N−H covalent bond in the N*ϵ*1^a^ species that experiences stronger hydrogen bonding.^[67]^ Indeed, the N*ϵ*1^a^ atom would preferentially hydrogen bond to a water molecule, represented by an intense correlation with ^1^H at 4.7 ppm (this shift in accordance with the reported ^1^H data for the structural water in the canonical Cph1 phytochrome^[68]^ and in CBCR AnPixJg2^[42]^). These results would suggest that the N*ϵ*1^a^ indole is now released from the stacking with ring ***D*** via the rotation around the *χ*_1_,*χ*_2_ angles, thus disrupting the hydrogen bond with the ***A***‐ring carbonyl group but binding to a nearby water molecule instead. The detection of water contact for the N*ϵ*1 indole nitrogen is inconsistent with the crystal and NMR structures available for the red/green CBCRs in their respective dark states,[^23^, ^24^, ^25^] but supported by the molecular dynamics study of AnPixJg2, in which a central water molecule is persistent in the chromophore‐binding pocket (>1 μs) and is strategically‐placed within the hydrogen‐bonding distances to both the ***A***‐ring carbonyl and the W496‐homologous residue.^[34]^

Unlike the dark state, the HetCor spectrum of the Pg photoproduct contains only a single N*ϵ*1 species resonating at 130.7 ppm (Figure 6, in green). This observation clearly indicates a greater structural rigidity of W496, consistent with the refolding of a disordered loop region containing this Trp (residues 481–498 between *β*_3_ and *α*_3_) into a two‐turn helix (*α*_2’_) upon photoproduct formation (Figure 1b). Moreover, in the photoproduct, only three interfacial contacts involving N*ϵ*1 are resolved. The loss of the interactions is associated with the outward movement of W496 which is now extruding towards bulk solvent, and has therefore less ^1^H contacts with the neighboring residues (Figure 1b). Specifically, there are two aliphatic ^1^H contacts present below 3 ppm which can be assigned provisionally to the adjacent H*β* atoms of C528 and D531 at the distances of 3.6 and 3.3 Å, respectively, to the N*ϵ*1 atom (Figure 6). These results provide evidence of a structurally homogeneous W496 in the photoproduct, despite a net loss of interactions at this site with the surrounding environment. The outward positional shift of W496 allows exposure of the chromophore to solvent (Figure 6), resulting in a more solvated binding pocket,^[42]^ similar to the situation seen in the NpR6012g4 photoproduct.^[24]^ In this context it is interesting to note that the ultrafast dynamics of the WT photoproduct is heterogeneous,^[15]^ most likely associated with structural heterogeneity of the chromophore as revealed by our recent NMR studies.^[22]^ These findings, together with the present results on W496, suggest that in the photoproduct this Trp residue cannot be related to the pronounced heterogeneity of the kinetics observed in slr1393g3.

### Indole N−H Bond Lengths and Hydrogen‐bond Formation in the Rotameric Structures of W496 in the slr1393g3 Dark State

**Figure 7:**
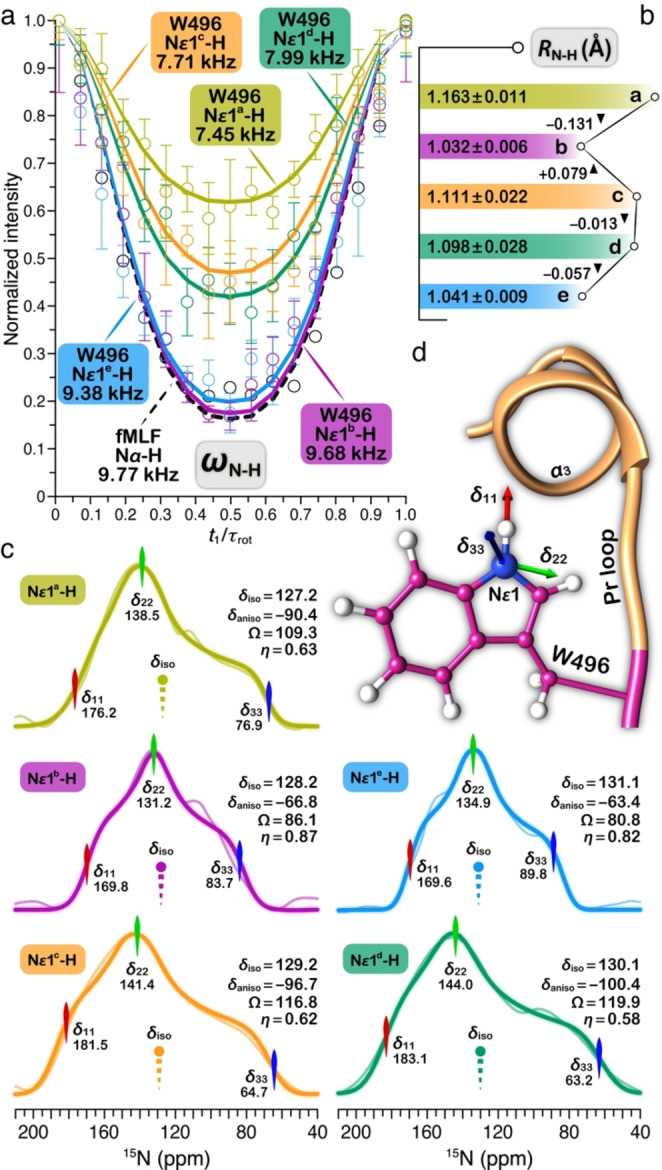
^15^N–^1^H dipolar couplings and ^15^N chemical shift anisotropies of W496N*ϵ*1 in the indole conformers. a) Dipolar dephasing curves for W496N*ϵ*1 in the five conformers were extracted from the *t*_1_ dimension of pseudo‐2D DIPSHIFT spectra of quadruply Y^2^F^2^‐substituted slr1393g3 variant sample in the Pr state. Dipolar couplings (*ω*_N‐H_) are labeled and experimental error bars were determined from the noise level. The chemotactic tripeptide *N*‐formyl‐methionyl‐leucylphenylalanine (fMLF) was used as rigid limit (black dashed curve). b) The resulting N*ϵ*1‐H bond lengths (*R*_N‐H_) in the five conformers. c) Cross sections of the five conformers were extracted from the 2D SUPER spectra (thin lines) with best‐fit simulations (thick lines). For each conformer, the isotropic value (*δ*_iso_ in ppm), anisotropy parameter (*δ*_aniso_ in ppm, describing the largest separation from the center of gravity of the lineshape), span (Ω in ppm, describing the maximum width of the powder pattern), and asymmetry parameter (*η*, indicating how much the lineshape deviated from that of an axially symmetric tensor) are indicated. The best‐fit uncertainties are ±2.1 ppm for *δ*_aniso_ and ±0.09 for *η*. d) Orientation of the principal axes on the molecular frame of W496N*ϵ*1 is depicted.[^71^, ^73^]

To further probe hydrogen‐bond formation of the W496 indole group in various rotameric structures, we performed a separated local field NMR experiment named dipolar chemical shift correlation (DIPSHIFT)[^69^, ^70^] to obtain indole N*ϵ*1‐H dipolar coupling at 293 K (Figure 7a). Previous NMR studies on cold shock protein embedded in trehalose confirmed that the dipolar couplings are insensitive to temperature near ambient.^[71]^ Hydrogen‐bond formation stretches the N−H bond from its covalent length, thus attenuating dipolar couplings.^[70]^ The two resonances assigned to N*ϵ*1^f^‐H and N*ϵ*1^g^‐H groups are at the noise level in the 2D ^15^N–^1^H DIPSHIFT spectrum, implicating minority conformations of W496 (consistent with the low intensities of the correlations involving N*ϵ*1^f^ and N*ϵ*1^g^ in the ^15^N–^1^H HetCor spectrum, Figure 6). The dipolar couplings of these two subpopulations are therefore not extracted from DIPSHIFT data. The other five N*ϵ*1‐H groups in the dark state show different N−H dipolar couplings which are easily identified from the depth of the dephasing curves (Figure 7a). Among them, the N−H couplings of N*ϵ*1^b^‐H and N*ϵ*1^e^‐H reach the rigid limit of 9.77 kHz (determined using a crystalline model tripeptide, fMLF), implicating the absence of motion. The corresponding N−H bond lengths are of 1.032 and 1.041 Å (Figure 7b), consistent with an unstretched bond determined as 1.029 Å for the amide N−H groups of fMLF. This result does not support the presence of hydrogen bond involving the W496 indole ring in these rotameric structures. In contrast, the N*ϵ*1^c^‐H and N*ϵ*1^d^‐H species showed stretched bonds of 1.111 (7.71 kHz) and 1.098 Å (7.99 kHz), respectively, implicating hydrogen bonding and corroborating the ‘Trp‐in’ conformation adopted by these two subpopulations. The most probable hydrogen‐bond acceptor for the indole ring in the two forms is the ***A***‐ring carbonyl because of the observed interfacial contacts to the mainchain H*β* protons of N532 and D498 (Figure 6) that are located adjacent to ring ***A*** (Figure 1b). A further reduced N−H coupling of 7.45 kHz was obtained for the N*ϵ*1^a^‐H species, corresponding to a significantly stretched bond of 1.163 Å (Figure 7a and b) which provides an explanation for the apparent absence of intermolecular contact between N*ϵ*1^a^ and its bonded H*ϵ*1^a^ proton (Figure 6). The large amplitude of bond elongation observed here is surprising. It may arise through hydrogen‐bond with water rather than with the ***A***‐ring carbonyl as seen in N*ϵ*1^c^‐H and N*ϵ*1^d^‐H groups. Proximity of the water protons to N*ϵ*1^a^ is derived from the strong HetCor correlation with a ^1^H resonance at 4.7 ppm (Figure 6). In the same spectrum, the H529 imidazole ring which provides hydrophobic packing contacts for the ***B***–***C*** plane from the *α*‐face is also seen to contact N*ϵ*1^a^. Taken together, these results imply that the W496 indole in this rotameric structure points away from the ring ***A*** but is rotated inward to face the two inner rings. The indole rotamerization would disrupt the π‐stacking with ring ***D***, promoting conformational freedom of the N*ϵ*1^a^‐H group.

A more complete picture on the local electronic environment, segmental dynamics, and hydrogen bonding is provided by the ^15^N anisotropic chemical shifts of the W496 indole ring in different rotameric conformations.[^72^, ^73^, ^74^, ^75^] We measured the ^15^N CSA values of W496N*ϵ*1^a–e^ using a method termed SUPER (separation of undistorted powder patterns by effortless recoupling),^[76]^ where the quasi‐static CSA powder patterns are recoupled in the indirect dimension as shown in Figure 7c. Like the DIPSHIFT analysis (Figure 7a), N*ϵ*1^f^ and N*ϵ*1^g^ were not taken into account for lineshape evolution because of the low intensities. Three principal values (*δ*_ii_, i=1, 2, and 3) were obtained by fitting the recoupled ^15^N CSA powder patterns using the SIMPSON program,^[77]^ from which the anisotropy parameter *δ*_aniso_, the asymmetry parameter *η*, and the span of CSA (Ω) can be calculated. Referring to the calculated molecular frame (Figure 7d), the spatial orientations of the shift tensors were assigned. Specifically, *δ*_11_ is oriented along the radial direction of the N−H bond, and the other two components, *δ*_22_ and *δ*_33_ are tangential to and perpendicular to the indole ring, respectively.

The ^15^N CSA span (Ω) is sensitive to the rigidity of N−H group. For example, the N*ϵ*1 nitrogen in the N*ϵ*1^c^‐H and N*ϵ*1^d^‐H groups have the span (116.8 and 119.9 ppm) that are significantly larger than those of the N*ϵ*1^b^‐H and N*ϵ*1^e^‐H nitrogens (86.1 and 80.8 ppm) and also the N*ϵ*1^a^‐H nitrogen (109.3 ppm). These results implicate the N*ϵ*1^b^‐H and N*ϵ*1^e^‐H species as the most rigid indole structures of W496, consistent with retention of both stacking and the hydrogen bond with the chromophore in the dark state, resembling the crystal structure (Figure 1b). The N*ϵ*1^a^‐H group is less rigid than N*ϵ*1^c^‐H and N*ϵ*1^d^‐H but more rigid than the N*ϵ*1^b^‐H and N*ϵ*1^e^‐H groups from the now freed W496 indole. This may arise due to the presence of hydrogen bond, for example, with a water molecule which is found to be proximal to N*ϵ*1^a^ (Figure 6). This interpretation is consistent with characterization of the asymmetry parameter *η* of the N*ϵ*1^a^ nitrogen (0.63) which is comparable to those of N*ϵ*1^c^ and N*ϵ*1^d^ (0.62 and 0.53) but smaller than those of the N*ϵ*1^b^ and N*ϵ*1^e^ nitrogens with the unstretched N−H covalent bond (0.87 and 0.82, Figure 7c). Moreover, the principal value *δ*_11_, assigned to be aligned along the N−H bond (Figure 7d), is a sensitive probe of hydrogen‐bond formation.^[70,72,73][]^ We found N*ϵ*1^c^‐H and N*ϵ*1^d^‐H to exhibit the most downfield *δ*_11_ values (Figure 7c), indicating that they were involved in the strongest hydrogen bond among all indole structures. For N*ϵ*1^a^‐H, however, *δ*_11_ shifts upfield by ∼6.0 ppm due to the effects of hydrogen bonding (with a water molecule) on the N−H bond stretching and the dipolar moment. Attenuation of the anisotropy parameter *δ*_aniso_ of N*ϵ*1^a^ as compared to those of N*ϵ*1^b^ and N*ϵ*1^e^ (by at least 6.3 ppm) provides further support for the different choice of hydrogen‐bonding partners of these indole rotamers, arising for example from different steric requirements.

### Determination of the Relative Content of Indole Rotameric Populations in W496 — The Minority Conformations (20 %) Retaining the π‐stacking and Hydrogen‐bonding Interactions with the Chromophore in the Dark State

**Figure 8:**
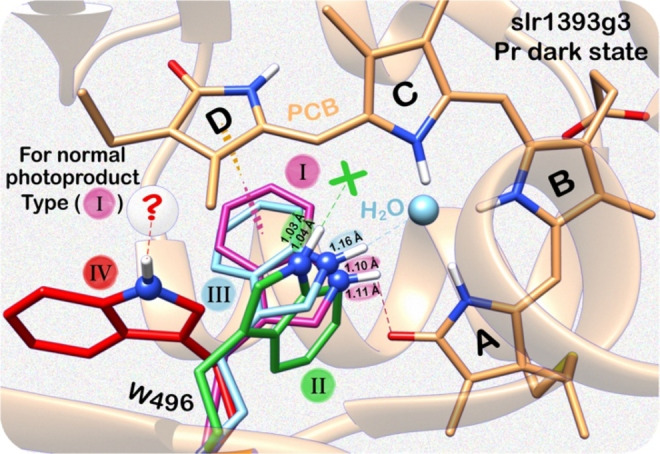
Proposed indole rotameric heterogeneity of W496 in the slr1393g3 dark state. Four types of sidechain rotameric conformations with different local interactions are shown. The N*ϵ*1‐H bond lengths obtained in this work are given. Sidechains retaining the stacking and hydrogen‐bonding interactions with the chromophore (type I), are known to undergo complete photoconversion. The hydrogen‐bonding partner of the N*ϵ*1 atom in the ‘Trp‐out’ structure (type IV) is not identified, as indicated by the question mark.

We identified a set of seven indole rotameric conformations of W496 in the dark state via the ^1^H contacts of N*ϵ*1 nitrogen (Figure 6). They fall into two major groups depending on whether they retain or disrupt the hydrogen bonding to the chromophore. The former is represented by the N*ϵ*1^c^‐H and N*ϵ*1^d^‐H species (denoted as type I, Figure 8), analogous to the ‘Trp‐in’ conformation of the W496‐homologous residue in the NpR6012g4 dark state which is known to undergo complete photoconversion yielding a normal photoproduct.^[24]^ The latter group can be divided into three subgroups based on the local environment and potential hydrogen‐bonding interactions of the indole nitrogen atom (types II–IV, Figure 8). Specifically, multiple independent lines of evidence from ^15^N–^1^H correlations, N−H bond lengths, and ^15^N CSA parameters (Figures 6 and 7) collectively suggest the absence of hydrogen bond in the N*ϵ*1^c^‐H and N*ϵ*1^d^‐H species (type II, Figure 8), with an unstretched bond of ~1.03 Å. By contrast, the prominent N*ϵ*1^a^‐H bond stretching (1.163 Å) is in excellent agreement with the presence of a strong N*ϵ*1^a^–H_2_O contact (Figure 6), indicative of hydrogen‐bond formation (type III, Figure 8). The type IV comprises the N*ϵ*1^f^‐H and N*ϵ*1^g^‐H species (Figure 8). Slices matching the two nitrogens exhibited only two interfacial contacts to a single aliphatic proton at *δ* ^H^ of 1.5 ppm (Figure 6), suggesting disruption of stacking between W496 and the ring ***D*** and the absence of a hydrogen bonding to either the chromophore (as in type I) or the structural water (type III). This conformation may be viewed as the ‘Trp‐out’ structure of NpR6012g4,^[24]^ in which only the H*β* proton of the conserved Asp (D498 in slr1393g3) sidechain adopting a ‘horizontal’ orientation can be found within 3.5 Å to the N*ϵ*1 atom, permitting interpretation of the weak correlation peaks in the N*ϵ*1^f^ and N*ϵ*1^g^ slices with ^1^H chemical shift of 1.5 ppm (Figure 6). The same spectrum also contained one more correlation from the H*β* proton of D498 to N*ϵ*1^d^ (from type I) but with ^1^H shift of 2.1 ppm. The dramatic ^1^H downfield change of 0.6 ppm could actually reflect an alternate sidechain conformation of D498 in this type, consistent with the ‘vertical’ orientation in the ‘Trp‐in’ structure of NpR6012g4.

**Figure 9:**
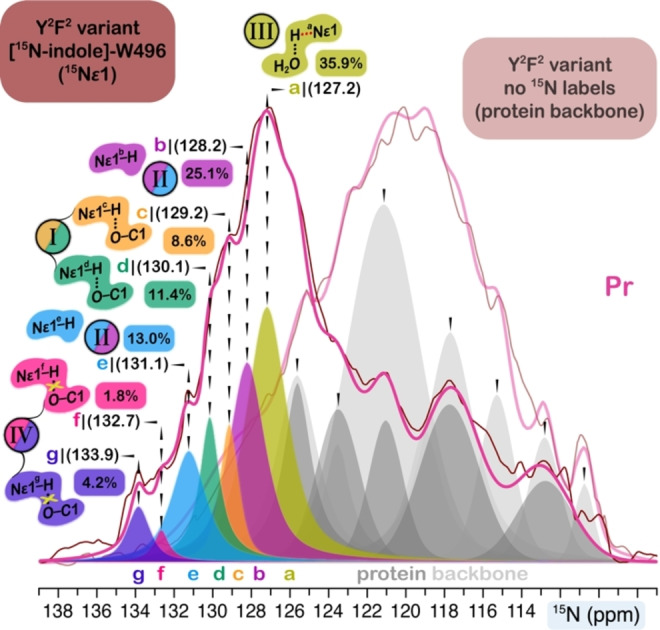
Four types of W496N*ϵ*1‐H groups in the slr1393g3 dark state and their relative populations. Stacked ^15^N NMR spectra of the [^15^N‐indole]‐labeled W496 (^15^N*ϵ*1) in the Y^2^F^2^ variant (maroon) and the corresponding counterpart sample at natural abundance (faded maroon) in their respective Pr states. Fitting spectra are colored dark and faded pink for the labeled and unlabeled samples, respectively. *δ* ^N^ of individual Voigt fits are labeled (see also Figure 6). Relative area in percentage of each Voigt fit (shaded and colored individually) was labeled by numbers. Fitting components of the labeled Y^2^F^2^ variant spectrum are shaded as dark gray. The corresponding fitting components in the spectrum of the unlabeled variant are shaded in light gray.

We identified four types of sidechain rotameric structures of W496 in the dark state, however, their relative content is not yet known. By integration of the ^15^N resonances in the 1D CP/MAS spectrum (Figure 9), these species could be quantified with a satisfactory approximation.^[39]^ Due to the severe spectral overlap of the N*ϵ*1 resonances of W496 and those originated from the amide nitrogens of the protein backbone in natural abundance, the ^15^N spectrum of the corresponding unlabeled Y^2^F^2^ sample was also recorded to ascertain the number of sidechain rotamers attained by the indole (Figure 9). Spectral fitting analysis of both slr1393g3 Y^2^F^2^ variant spectra corroborated a set of seven N*ϵ*1 resonances (N*ϵ*1^a^–N*ϵ*1^g^), matching the ^15^N slices in the HetCor spectrum (Figure 6). Resonances were directly quantified by a Voigt lineshape spectral model function,^[78]^ yielding the relative content between 1.8 and 35.9 % for the seven maxima ranging from 127.2–133.9 ppm (Figure 9). Surprisingly, the type I rotamers, assigned as the population undergoing full photoconversion sum up to only 20 %, remarkably lower than that of ~90 % in the NpR6012g4 dark state.^[24]^ The population difference enables us to, at least partially, explain the low quantum yield of 8 % for the Pr → Pg reaction in slr1393g3,[^15^, ^52^] but in contrast being as high as 40 % for that of NpR6012g4.^[36]^ The type IV conformation, having the ‘Trp‐out’ orientation was seen in 6 % of the rotameric structures, similar to that of the ‘Trp‐out’ population in NpR6012g4 (~10 %).^[24]^ The other two types of rotameric structures (II and III) account for 38.1 and 35.9 %, respectively, however, having no equivalents in NpR6012g4. Therefore, additional stabilizing effects are required for formation of the latter rotameric types in slr1393g3, e. g., a water molecule serving as the hydrogen‐bonding partner to stabilize the type III conformation (Figure 8) that is absent in the immediate neighborhood of the lid Trp residue in NpR6012g4. The structural difference between the two proteins is also evident by the introduction of His in place of the lid Trp which produces two dark‐state side populations (orange‐ and red‐ absorbing at 573 and 680 nm, respectively, Figure 2a) in slr1393g3, but fails to restore the chromophore binding in NpR6012g4.^[51]^

## Conclusions

Besides the determinative role of the central lid Trp in slr1393g3 (W496) for formation and stabilization of the dark state, we have demonstrated the structural heterogeneity at this Trp residue in the dark state using solid‐state NMR experiments on a TG‐incorporated, quadruply Trp‐deleted Y^2^F^2^ variant sample with the remaining W496 ^15^N‐indole labeled. The indole ring exhibited four types of rotameric structures with distinct local electronic environment, segmental dynamics, and hydrogen‐bonding interactions. Moreover, the quantitative analysis based on the ^15^N resonance integration determined the relative content of these rotameric populations. The type I species, adopting the ‘Trp‐in’ conformation turns out to be only 20 %. This proportion, however, is much lower than that estimated for the NpR6012g4 dark state of ~90 % which is known to undergo full photoconversion.^[24]^ Such a difference can be correlated directly to the unusually low quantum yield of 8 % for the photoproduct formation in slr1393g3, being at least three‐ to four‐fold lower in NpR6012g4 and the related red/green CBCRs. Other types of indole rotameric conformations for W496 are likely to have little or no capacity to yield normal photoproduct, in which additional stabilizing effects provided by, e. g., a (solvent) water molecule proximal to W496, are identified. It is thus plausible that slr1393g3 has a more disordered, solvated binding pocket in the dark state than that of NpR6012g4, despite their sequential and structural homology and their common absorption features.[^24^, ^25^] In addition to providing new insights into rotameric heterogeneity of W496, our studies highlight the importance of its interactions with the chromophore (electrostatic and hydrogen‐bonding), in that compromising any one would lead to a reduced forward quantum yield in slr1393g3. Mutations that facilitate retention of the W496–chromophore interactions, e. g., prohibition of the influx of water molecules into the binding pocket may enhance the quantum yield, promoting development of CBCRs for biotechnological applications. It is worth noting here that although Trp is the least frequent amino acid in proteins (due to its energy demanding biosynthesis), it often plays a fundamental role in the function of proteins, notably in the interaction with embedded ligands[^79^, ^80^] or, here, with covalently bound chromophores in photoreceptors. The method applied in this work, i. e., the selective ^15^N‐labeling of a single Trp residue, precisely demonstrates its pivotal function for the photoisomerization of the bilin chromophore in this bacterial class of phytochromes. This approach can be seen as proof of principle for probing the function of Trp residue at the atomic level upon employment of solid‐state NMR spectroscopy.

## Conflict of Interests

The authors declare no conflict of interest.

1

## Supporting information

As a service to our authors and readers, this journal provides supporting information supplied by the authors. Such materials are peer reviewed and may be re‐organized for online delivery, but are not copy‐edited or typeset. Technical support issues arising from supporting information (other than missing files) should be addressed to the authors.

Supporting Information

## Data Availability

Research data are not shared.
