# Supplementary material for: Rotameric Heterogeneity of Conserved Tryptophan Is Responsible for Reduced Photochemical Quantum Yield in Cyanobacteriochrome Slr1393g3
Source: Chemphyschem. 2024 Nov 12;26(2):e202400453. doi: 10.1002/cphc.202400453 (PMC11733413; doi:10.1002/cphc.202400453)
Supplement: Supplementary file 1 — Supporting Information [file CPHC-26-e202400453-s001.pdf]

ChemPhysChem

Supporting Information

# Rotameric Heterogeneity of Conserved Tryptophan Is Responsible for Reduced Photochemical Quantum Yield in Cyanobacteriochrome Slr1393g3

Lisa Köhler, Florian Trunk, Valentin Rohr, Tobias Fischer, Wolfgang Gärtner, Josef Wachtveitl, Jörg Matysik, Chavdar Slavov,* and Chen Song*

Supporting Information
©Wiley-VCH 2019
69451 Weinheim, Germany

# Rotameric Heterogeneity of Conserved Tryptophan Is Responsible for Reduced Photochemical Quantum Yield in Cyanobacteriochrome Slr1393g3

Lisa Köhler,[a] Florian Trunk,[b] Valentin Rohr,[a] Tobias Fischer,[b] Wolfgang Gärtner,[a] Josef Wachtveitl,[b] Jörg Matysik,[a] Chavdar Slavov,*[b,c] and Chen Song*[a]

[a] Dr. L. Köhler, V. Rohr, Prof. Dr. W. Gärtner, Prof. Dr. J. Matysik, Dr. C. Song
Institut für Analytische Chemie
Universität Leipzig
04103 Leipzig (Germany)
E-Mail: chen.song@uni-leipzig.de (C. Song)

[b] Dr. T. Fischer, F. Trunk, Prof. Dr. J. Wachtveitl, Dr. C. Slavov
Institut für Physikalische und Theoretische Chemie
Goethe-Universität Frankfurt
60438 Frankfurt am Main (Germany)

[c] Dr. C. Slavov
Department of Chemistry
University of South Florida
33620 Tampa (United States of America)
E-Mail: chslavov@usf.edu (C. Slavov)

## Experimental Procedures

### *Protein expression and purification*

All variants of slr1393g3 were generated by PCR using the plasmid pET30 encoding slr1393g3 from *Synechocystis* as a template[1] and the degenerate primers listed in Table S1. Subsequent to *DpnI* digestion, the PCR products were transformed into *E.coli XL10Gold* competent cells. After plasmid isolation and sequence identification, the plasmids as well as the plasmid pACYC carrying the PCB-generating enzymes *ho1-pcyA* were transformed into *E.coli BL21 DE3* competent cells to allow heterologous protein expression and *in vivo* assembly of the phytochrome. A detailed description of the protein expression, purification and sample preparation for solid-state NMR application is given elsewhere.[2] The site-specific isotope labeling of the Trp indole sidechain was accomplished by adding $^{15}N$-labeled indole to the M9 growth medium prior to protein expression.[3]

Table S1. DNA sequence of the degenerated primers used in this study.

| Primer | Sequence | Position |
|---|---|---|
| P1 | 5’ ccggactWtagcggggaatttatccatg 3’ | W470F/Y |
| P2 | 5’ cccgctaWagtccggattaaagcgatag 3’ | |
| P3 | 5’ ccaaatgtWtgaaccactgaaagacttgc 3’ | W483F/Y |
| P4 | 5’ gtggttcaWacatttgggccaccg 3’ | |
| P5 | 5’ ccccctcYWTcaagacacctatctccagg 3’ | W496H/L/Y |
| P6 | 5’ gtgtcttgaWRgagggggaaattattttgc 3’ | |
| P7 | 5’ caactctWtggcctgttgggggc 3’ | W553F/Y |
| P8 | 5’ caggccaWagagttgttcccccacaaac 3’ | |
| P9 | 5’ ccccgccattWtcaagcgagggaaatcc 3’ | W567F/Y |
| P10 | 5’ ctcgcttgaWaatggcggggagcc 3’ | |

### *Preparation of trehalose glass (TG) samples*

The slr1393g3 $Y^2F^2$ variant was dialyzed against 20 mM Tris, 20 mM NaCl at pH 7.2, and then concentrated using an ultracentrifugation filter (Merck Millipore, MWCO 10000). For NMR use, the appropriate volume of trehalose (1.2 M stock solution), dissolved in 20 mM Tris, 20 mM NaCl, pH 7.2, was mixed with the slr1393g3 $Y^2F^2$ to achieve a molar ratio of 50:1 T/P. The formulation was dried on a Petri dish under continuous $N_2$ gas flow and under continuous illumination with light of 535 nm for Pr dark state or 650 nm for Pg photoproduct. The slr1393g3 $Y^2F^2$ TG samples were then characterized by UV-vis spectroscopy, crushed gently and loaded into a 3.2-mm $ZrO_2$ rotor. Approximately 118 mg TG as Pr and 129 mg as Pg were used in this study. No additional illumination was applied during the NMR acquisition.

### *UV-Vis absorbance spectroscopy*

All measurements were performed on a Shimadzu 1900i spectrophotometer equipped with the temperature regulation system TCC-240A. Photoreversibility of slr1393g3 WT and variants in solution was confirmed by the irradiation protocol of 650 → 530 → 650 nm (with FWHM of ~17.3 nm) using 60mW LEDs (Roithner Laser Technik) at 296 K for two minutes. Moreover, the spectroscopic properties as well as the stability of the $Y^2F^2$ variant sample embedded in TG were characterized by UV-vis spectroscopy prior to the acquisition of NMR experiments.

### *MAS NMR spectroscopy*

All $^{15}N$ 1D CP, 2D $^{15}N$–$^{1}H$ HetCor, $^{15}N$ 2D SUPER and $^{15}N$–$^{1}H$ 2D DIPSHIFT experiments were performed on a Bruker AVANCE-III 600MHz NB NMR spectrometer (Rheinstetten, Germany) equipped with a 3.2 mm double-resonance MAS probe. The experiment temperature was set to 296K and was maintained with a deviation of ± 0.2 K by a temperature control unit. MAS spinning rates of 15000 ± 5 Hz for the $^{15}N$ CP and HetCor experiments were controlled by a Bruker MAS unit. Optimized $^{1}H$ and $^{15}N$ $\pi/2$ pulse lengths were 2.3 and 3.6 s, respectively. $^{15}N$ transverse magnetization created by ramped CP (70–100%) was transferred from $^{1}H$ with an optimal contact time of 2 ms. An r.f. lock field of 39.3 kHz was applied on $^{15}N$, fulfilling the Hartmann–Hahn condition. During the acquisition, a swept-frequency two-pulse phase modulation heteronuclear decoupling ($SW_f$-TPPM) at a $^{1}H$ r.f. field of 113.2 kHz was used for $^{1}H$ decoupling. For all $^{15}N$ CP spectra of the protein samples, 24576 scans were accumulated with a relaxation delay time of 2.0 s. A line broadening of 20 Hz and zero-filling to 8192 points was used prior to Fourier transformation. $^{15}N$–$^{1}H$ HetCor experiments were carried out using frequency-switched Lee–Goldburg $^{1}H$ homonuclear dipolar decoupling (FSLG). The $^{1}H$ chemical shift scaling factor was determined experimentally to be 0.57.[4] All experiments were acquired with an optimized CP contact time of 2 ms, 60 increments in the indirect dimension and 1984 or 2656 scans for each increment for the dark-state and the photoproduct TGs, respectively. The relaxation delay was 2.0 s. A 90° shifted squared sine-bell window function (SSB = 4) and zero-filling to 1024 points was applied to the indirect dimension. A 90° shifted squared sine-bell window function (SSB = 2) was applied in the direct dimension and further zero-filled to 4096 points. The spectral width divided by the scaling factor yielded the appropriate scaling of the $^{1}H$ indirect dimension. The spectra were externally referenced to the correlation 176.3/12.6 ppm ($^{15}N/^{1}H$) of solid L-Histidine·HCl in its cationic form.[5] The SUPER experiment was acquired at a spinning frequency of 5000 ± 5 Hz resulting in a r.f. field of 42.4 kHz for CSA recoupling. A total of 24 $t_1$-increments were recorded, accumulating 4096 scans during each of the two $\gamma$-integral points with a recycle delay of 2.0 s. A spectral width of 18245 Hz was used. The offset was set to 100 ppm. During the acquisition a $SW_f$-TPPM heteronuclear decoupling was applied. All CSA cross-sections from the 2D SUPER experiment were fitted using SIMPSON. The DIPSHIFT experiment was acquired at a spinning frequency of 3500 ± 5 Hz. In total, 17 $t_1$-increments were recorded, accumulating 1472 scans each. A CP contact time of 2 ms was used. Windowed phase-modulate Lee–Goldburg[6] homonuclear decoupling (m5m-shape from the Bruker library) was applied during the evolution time with 98.9 kHz r.f. amplitude. During the acquisition a $SW_f$-TPPM heteronuclear decoupling was applied. The data was zero-filled to 8192 points prior to Fourier transformation and an exponential window function was used. All DIPSHIFT dephasing curves were fitted using SIMPSON. Here, the scaling factor of 0.5 arising from the PMLG decoupling was considered. The tripeptide *N*-formyl-methionyl-leucyl-phenylalanine (fMLF) was used to experimentally determine the rigid limit. $^{15}N$ chemical shift was referenced to the $NH_4^+$ signal of solid $^{15}NH_4NO_3$ at 23.5 ppm. The data was processed with Bruker Topspin 4.1.3 and further analysed with MestReNova 14.1.0 (Mestrelab Research, Santiago de Compostella, Spain).

## *Fluorescence spectroscopy*

Fluorescence spectra were measured with an FP 8500 fluorimeter (Jasco) and corrected for background, reabsorption, detector sensitivity, solvent contributions, and the scattering of the excitation. All measurements were averaged over 5 scans with an illumination period between all scans to avoid photoproduct accumulation (Fig. S1).

## *Ultrafast transient absorption spectroscopy*

The time-resolved transient absorption measurements in the visible spectral range were recorded using a home-built pump-probe setup as described in detail previously.[7] In brief, the fundamental laser pulses (1 mJ, 775 nm, 150 fs, 1 kHz) provided by a Ti:Sapphire amplifier system (Clark, MXR-CPA-iSeries) were split into a pump and a probe pathway. The pump pulses were generated using a home-built two stage NOPA (noncollinear optical parametric amplifier)[8] with a prism compressor located in between the NOPAs for pulse compression. White light continuum probe pulses (300–750 nm) were generated by focusing the laser fundamental into a $CaF_2$ crystal (5 mm) which were split into a probe and reference beam before detection. The probe beam was focused at the sample position, collected and guided into a spectrograph (AMKO Multimode) containing gratings with 600 grooves/mm blazed at 500 nm and a photodiode array with the detection range set to 400–720 nm. The reference beam was guided directly into a second spectrograph of identical configuration. The instrument response function of ~60–80 fs was estimated from the pump probe cross correlation and confirmed using an autocorrelator (APE, APE Pulse Check). All measurements were carried out under magic angle conditions (54.7°

pump-probe polarization difference) to avoid anisotropic contributions. The sample was measured in a fused silica cuvette with an optical path length of 1 mm which was constantly being moved perpendicular to the direction of the probe pulse propagation to avoid photodegradation. Samples were excited with 45 nJ/pulse and irradiated continuously using a 530 nm or 660 nm high-power LED (ThorLabs, M530L4 or M660L4) to avoid the accumulation of the Pg photoproduct or Pr dark state, respectively.

### *UV-Vis flash photolysis*

The samples were excited by pump pulses produced by an optical parametric oscillator (OPO) (preciScan, GWU-Lasertechnik) pumped by a Nd:YAG laser (SpitLight 600, Innolas Laser). The OPO was adjusted to 640 nm for the Pr or to 530 nm for the Pg measurements with an average pulse energy of 2.4 ± 0.2 mJ/cm$^2$. A continuous wave Xenon lamp (Hamamatsu LC-8) was used to generate the probe light, combined with two matched monochromators, placed before and after the light had passed the sample. Changes in the absorbance were then detected by a photomultiplier tube (PMT) and the obtained signal was converted into an electrical signal and recorded using an oscilloscope (DPO5204B-10RL, Tektronix). In all measurements, the absorbance changes were recorded between 470 nm and 700 nm using a step size of 10 nm with at least 30 averaged scans per wavelength. Between each recorded scan, the sample was illuminated for a minimum of 6 s using a 530 nm or 660 nm LED (ThorLabs, M530L4 or M660L4) to avoid the accumulation of the Pg photoproduct or Pr dark state, respectively. To obtain feasible data sizes, the single transients were reduced by averaging data points to a combined linear and logarithmic timescale before kinetic analysis was performed.

## Data analysis

Analysis of the time-resolved data was performed using OPTIMUS (http://www.optimusfit.org).[9] All transient absorption datasets were corrected for the coherent artifact using a function composed of a Gaussian and its first and second derivatives.[10,11] Analysis of flash photolysis data was performed using global target analysis with a sequential model. The number of states equals the number of exponentials used in global analysis. The model is then fitted directly to the experimental data and yields the lifetimes of the observed states in a sequential kinetic scheme, the evolution-associated decay spectra (EADS) which display the spectral properties of each kinetic component/state complemented by the decay-associated spectra (DAS) displaying the spectral changes attributed to the lifetimes. In the context of flash photolysis, the DAS exhibits positive amplitudes to account for the decay of photoproduct absorption (PA) or the rise of ground state bleach (GSB). Negative amplitudes describe the rise of PA or the decay of GSB.

For the ultrafast datasets, we performed model-independent lifetime distribution analysis (LDA): a method that deals naturally with non-exponential kinetics such as heterogeneous or non-exponential dynamics. In LDA, the pre-exponential amplitudes of a quasi-continuous set (n = 100) of exponential functions with fixed, equally spaced (on a decimal logarithm scale ranging from 30 fs to 5000 ps) lifetimes were determined. These pre-exponential amplitudes at each detection wavelength are then plotted in the form of a contour lifetime density map (LDM). The reading of LDMs is similar to the reading of DAS, as described previously: Positive (red) amplitudes account for the decay of the excited state and product absorption (ESA and PA) or the rise of ground state bleach and stimulated emission (GSB and SE). Negative (blue) amplitudes describe the rise of ESA and PA or the decay of GSB and SE.

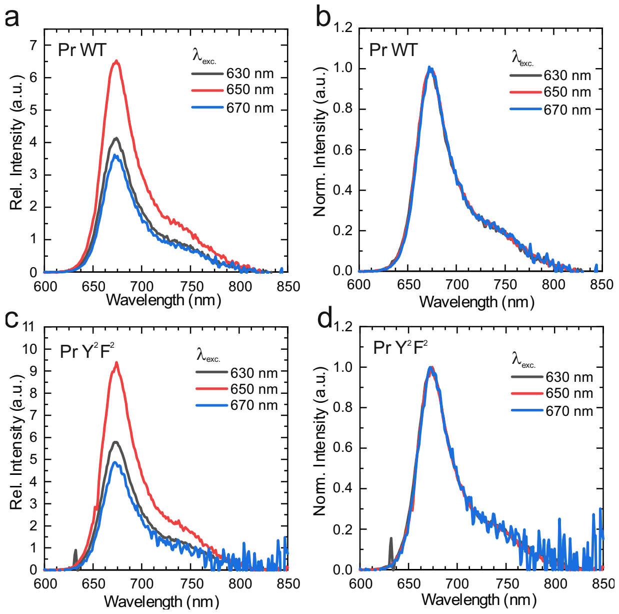


**Figure S1.** Excitation wavelength-dependent emission spectra of the slr1393g3 dark state for the WT and $Y^2F^2$. (*Top*, panels a-b) a) Emission spectra of the Pr WT excited at 630, 650 and 670 nm. b) Normalized emission spectra of the WT. (*Bottom*, panels c–d) c) Emission spectra of the Pr $Y^2F^2$ variant excited at 630, 650 and 670 nm. d) Normalized emission spectra of the $Y^2F^2$ variant. Note that the fluorescence is excitation wavelength independent and the same for $Y^2F^2$ variant and WT.

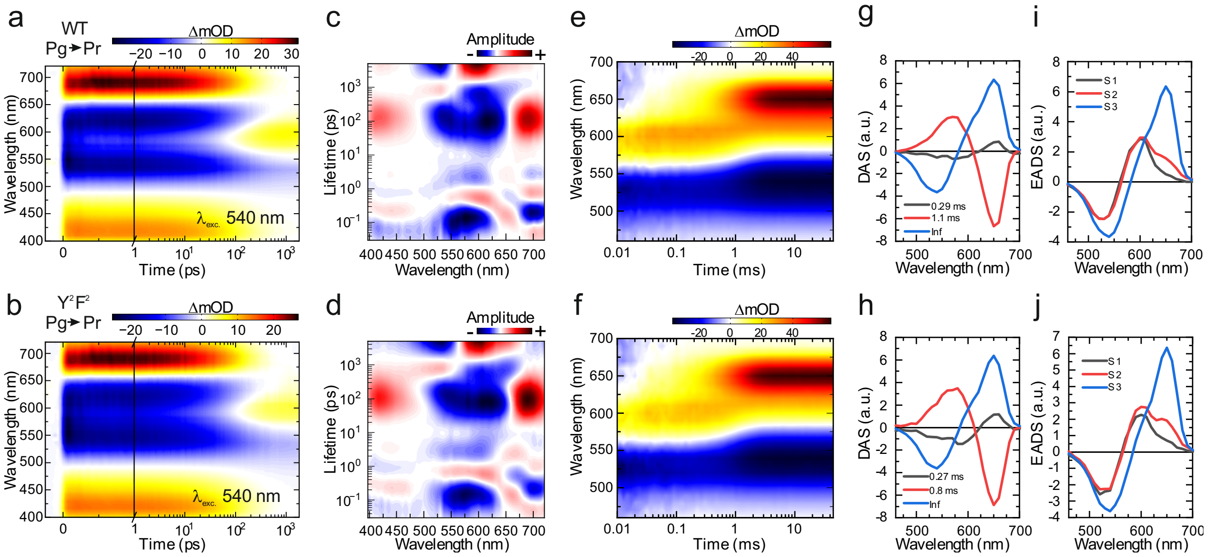


**Figure S2. Ultrafast excited-state and ms dynamics of the slr1393g3 quadruple $Y^2F^2$ variant.** (*Left*, panels *a–b*) Transient absorption data of the reverse (Pg → Pr*)* dynamics *of the* $Y^2F^2$ variant compared to those of the WT shown. *(Left/middle*, panels *c-d*) Corresponding lifetime density maps (LDMs) obtained from lifetime distribution kinetic analysis of the transient absorption data. The ultrafast data are shown after excitation of the Pg state at 540 nm. (*Middle*, panels *e–f*) ms forward dynamics of the $Y^2F^2$ variant compared to the WT. *(Right/middle*, panels *g–h*) corresponding decay-associated spectra (DAS). (Right, panels *i-j)* corresponding evolution-associated spectra (EADS). The ms dynamics were obtained after excitation of the Pg state at 530 nm.

The TA data and LDM of the slr1393g3 $Y^2F^2$ variant and WT exhibit high similarity (Fig. S2, a–d). A detailed description of the reverse reaction on the ultrafast timescale of the slr1393g3 WT has already been published.[12] In short, the transient absorption data consist of three positive absorption difference signals. The first two are present directly after excitation and located below 470 nm and above 650 nm and therefore assigned as ESA. The third, centered at 600 nm, raises on 100 ps timescale and is assigned to PA. Two negative signals are clearly resolved: the GSB at 540 nm and the SE located at 630 nm. The GSB of the variant shows a bathochromic shift compared to the WT and thus the GSB and the SE are not as spectrally separated in the variant. The LDMs from the LDA show five dominant lifetime components. The first is centered around 100 fs and associated with dynamics on the excited state potential energy surface. In the range above 1 ps and below 10 ps alternating positive and negative amplitudes are displayed in the LDM, therein the distribution has previously been associated with early photoproduct formation as well as partial GSB recovery. The third distribution is centered around 100 ps and with its positive and negative amplitudes located on the ESA and GSB/SE respectively describes the dominant decay of the ES. The fourth distribution is located at 300 ps peaking at 600 nm with a negative amplitude. This distribution is assigned to the formation of the photoproduct Lumi-Pg from a ground state intermediate. Lastly, the fifth distribution is found on the upper limit of the LDM displaying the remaining bleach around 530 nm and the PA centered around 580 nm. Like the ultrafast dynamics, the reverse reaction on the ms timescale is not majorly altered (Fig. S2, e–j) The data shows two positive bands and a negative signal, assigned to the bleach. The first positive signal absorbs around 580 nm and decays forming the Pr state, absorbing at 640 nm. The last transition, forming the Pr state, is slightly accelerated in the $Y^2F^2$ variant where the intermediate decays with a lifetime of 0.8 ms, while the lifetime in the WT is found to be 1.1 ms.

## References

[1] Y. Chen, J. Zhang, J. Luo, J.-M. Tu, X.-L. Zeng, J. Xie, M. Zhou, J.-Q. Zhao, H. Scheer, K.-H. Zhao, *FEBS J.* **2012**, *279*, 40–54.

[2] L. Köhler, W. Gärtner, J. Matysik, C. Song, *ChemPhotoChem* **2022**, *6*, e202100220.

[3] J. Schörghuber, T. Sára, M. Bisaccia, W. Schmid, R. Konrat, R. J. Lichtenecker, *ChemBioChem* **2015**, *16*, 746–751.

[4] B.-J. van Rossum, H. Förster, H. J. M. de Groot, *J. Magn. Reson.* **1997**, *124*, 516–519.

[5] S. Li, M. Hong, *J. Am. Chem. Soc.* **2011**, *133*, 1534–1544.

[6] E. Vinogradov, P. K. Madhu, S. Vega, *Chem. Phys. Lett.* **2002**, *354*, 193–202.

[7] C. Slavov, N. Bellakbil, J. Wahl, K. Mayer, K. Rück-Braun, I. Burghardt, J. Wachtveitl, M. Braun, *Phys. Chem. Chem. Phys.* **2015**, *17*, 14045–14045.

[8] E. Riedle, M. Beutter, S. Lochbrunner, J. Piel, S. Schenkl, S. Spörlein, W. Zinth, *Appl. Phys. B* **2000**, *71*, 457–465.

[9] C. Slavov, H. Hartmann, J. Wachtveitl, *Anal. Chem.* **2015**, *87*, 2328–2336.

[10] S. A. Kovalenko, A. L. Dobryakov, J. Ruthmann, N. P. Ernsting, *Phys. Rev. A* **1999**, *59*, 2369–2384.

[11] A. L. Dobryakov, S. A. Kovalenko, A. Weigel, J. L. Pérez-Lustres, J. Lange, A. Müller, N. P. Ernsting, *Rev. Sci. Instrum.* **2010**, *81*, 113106.

[12] C. Slavov, X.-L. Xu, K.-H. Zhao, W. Gärtner, J. Wachtveitl, *Biochim. Biophys. Acta Bioenerg.* **2015**, *1847*, 1335–1344.
